# Association of physical frailty with cardiac conduction disorder events in the UK Biobank

**DOI:** 10.3389/fcvm.2026.1967667

**Published:** 2026-10-19

**Authors:** Qian Tang, Jingting Lyv, Chengfeng Li, Meihui Wang, Guosheng Fu, Yao Wang, Lu Yu

**Affiliations:** 1 Department of Cardiology, Sir Run Run Shaw Hospital, Zhejiang University School of Medicine, Hangzhou, China; 2 Zhejiang Key Laboratory of Cardiovascular Intervention and Precision Medicine, Hangzhou, China; 3 Engineering Research Center for Cardiovascular Innovative Devices of Zhejiang Province, Hangzhou, China; 4 MOE Key Laboratory of Macromolecular Synthesis and Functionalization, Department of Polymer Science and Engineering, Zhejiang University, Hangzhou, China

**Keywords:** cardiac conduction disorder, fascicular block, left bundle branch block, physical frailty, UK Biobank

## Abstract

**Background:**

Practical markers for identifying individuals at risk of cardiac conduction disorders (CCDs) before overt disease are limited. Physical frailty reflects reduced physiological reserve and may capture vulnerability beyond chronological age and individual cardiometabolic factors. Herein, we investigated the association of physical frailty with incident CCD in the UK Biobank cohort.

**Methods:**

We included 423,162 participants with data for a modified Fried frailty phenotype and linked health-record follow-up after excluding those with cardiovascular disease, CCDs, or pacemaker implantation at baseline. Participants were classified as non-frail, prefrail, or frail. The primary outcome was incident CCD, and the secondary outcomes were severe conduction block and left bundle branch or fascicular block. Cox proportional hazards models estimated hazard ratios (HRs) and 95% confidence intervals (CIs).

**Results:**

Overall, 58.2% of participants were non-frail, 38.5% were prefrail, and 3.3% were frail; 18,067 incident CCD events occurred during the follow-up. Compared with participants who were non-frail, participants who were prefrail and frail were at higher risks of CCDs after multivariable adjustment [HR, 1.23 (95% CI, 1.19–1.27) and HR, 1.71 (95% CI, 1.60–1.83), respectively; *P* for trend <0.01]. The corresponding HRs were 1.13 (95% CI, 1.09–1.18) and 1.44 (95% CI, 1.32–1.59) for severe conduction block and were 1.24 (95% CI, 1.17–1.32) and 1.61 (95% CI, 1.43–1.81) for left bundle branch or fascicular block.

**Conclusions:**

Physical frailty was associated with graded increases in incident CCD and related conduction outcomes, with excess risk evident at the prefrailty stage. Frailty assessment may help identify individuals with increased susceptibility, although the observational design does not establish causality or demonstrate that modifying frailty would reduce risk.

## Graphical Abstract

**Figure:**
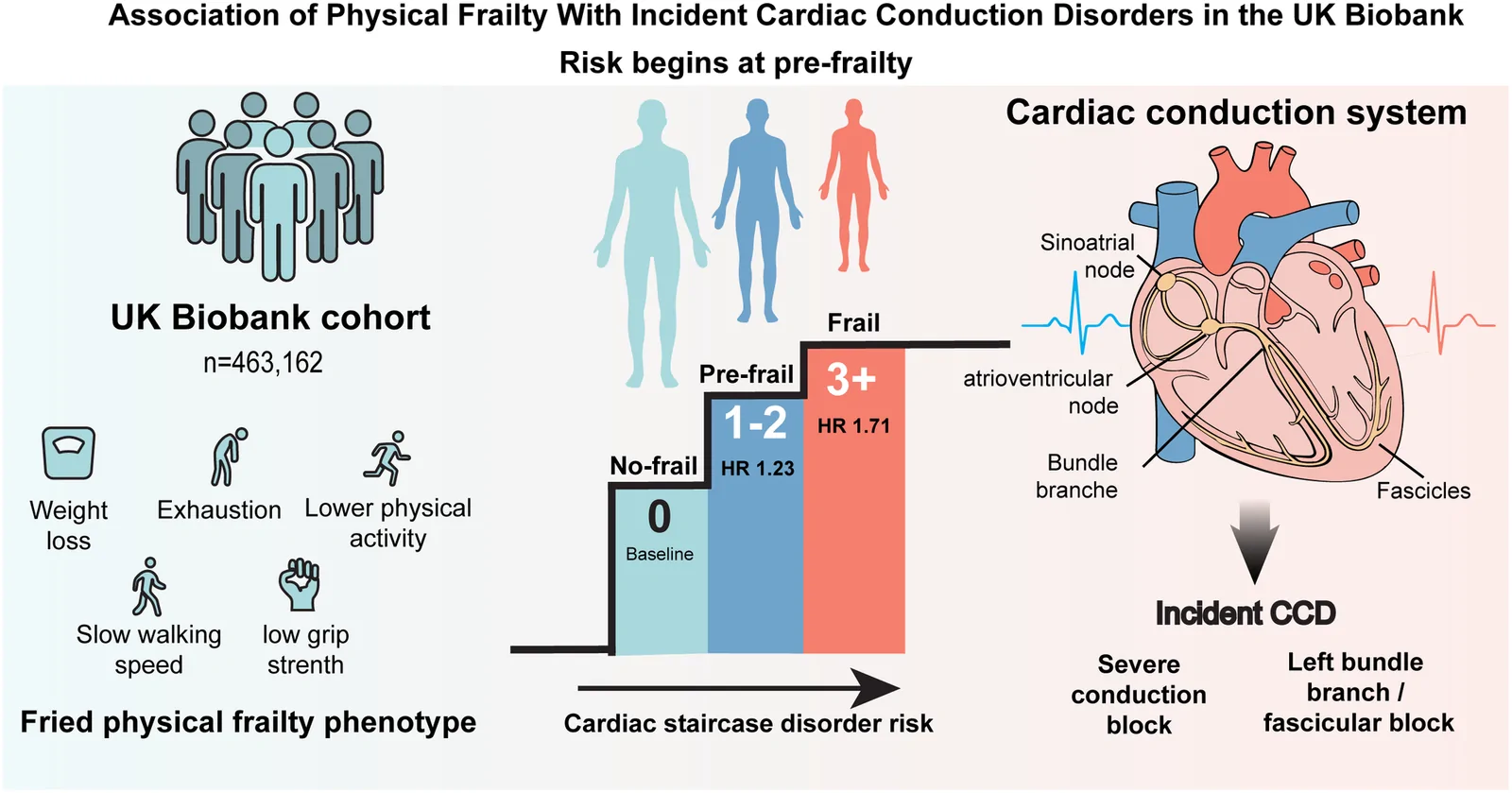

## Introduction

Cardiac conduction disorders encompass abnormalities of atrioventricular, bundle branch, fascicular, and intraventricular conduction (1, 2). Although some conduction findings are detected incidentally, clinically important conduction disease may cause symptoms, contribute to heart failure and adverse cardiovascular outcomes, and necessitate permanent pacemaker implantation (1, 2). Therefore, identifying individuals at increased risk before a clinically overt conduction disease develops remains clinically relevant (2).

Recent studies suggest that conduction disease may be influenced by systemic and potentially modifiable risk states (3–5). Better cardiovascular health, healthier lifestyle patterns, lower inflammatory burden, and markers of biological ageing each have been linked to conduction outcomes (3, 4, 6, 7). This evidence has shifted part of the field from descriptive electrocardiography toward prevention-oriented epidemiology (2). Population-based studies have further identified clinical risk factors for atrioventricular block and cardiac conduction block, including cardiometabolic traits such as obesity (8, 9).

Physical frailty is a clinical phenotype of reduced physiological reserve (10, 11). The Fried phenotype captures weight loss, exhaustion, low activity, slow walking speed, and weak grip strength (10). Recent UK Biobank analyses and cardiovascular cohorts suggest that frailty provides risk information across cardiovascular and cardiovascular–kidney–metabolic conditions (12–15). A previous UK Biobank study reported graded associations of physical frailty with several incident arrhythmia categories, including conduction system diseases (16). However, conduction system disease was evaluated as one component of a broader arrhythmia analysis, and clinically distinct conduction outcomes were not examined separately. Therefore, it remains unclear whether the magnitude of the association with frailty differs across overall CCD, advanced atrioventricular conduction disease, and bundle branch or fascicular conduction disorders.

Accordingly, we examined the association of physical frailty, assessed using a modified Fried phenotype, with incident CCD in 423,162 UK Biobank participants without cardiovascular disease (CVD), cardiac conduction disorder (CCD), or pacemaker implantation at baseline. In addition, we evaluated severe conduction block and left bundle branch or fascicular block as prespecified secondary outcomes. We hypothesized that progressively greater frailty would be associated with higher risks of incident CCD and its clinically relevant subtypes. Exploratory subgroup analyses were conducted to assess the consistency of these associations across characteristics observed in participants.

## Methods

### Data sources and study population

This prospective cohort study used data from the UK Biobank, a large population-based cohort that recruited more than 500,000 adults aged 40–69 years from 2006 to 2010 across the United Kingdom (17). At baseline, participants completed touchscreen questionnaires and nurse-led interviews and underwent physical measurements and biological sampling. Long-term health outcomes were ascertained through linkage to national hospital inpatient records, procedure records, and death registries (17). The UK Biobank received ethical approval from the North West Multicentre Research Ethics Committee (REC; reference no. 21/NW/0157), and all participants provided written informed consent. The present analysis was conducted with permission to use data of the UK Biobank (application no. 866163).

Participants were eligible if they had sufficient baseline data to derive the modified Fried frailty phenotype and had linked health-record follow-up. For the present analysis, participants with a history of CVD, CCD, or pacemaker implantation at or before baseline were excluded. After these exclusions, the final analytic cohort included 423,162 participants, comprising 246,284 non-frail, 162,868 prefrail, and 14,010 frail participants. Cohort construction is shown in Figure 1.

**Figure 1:**
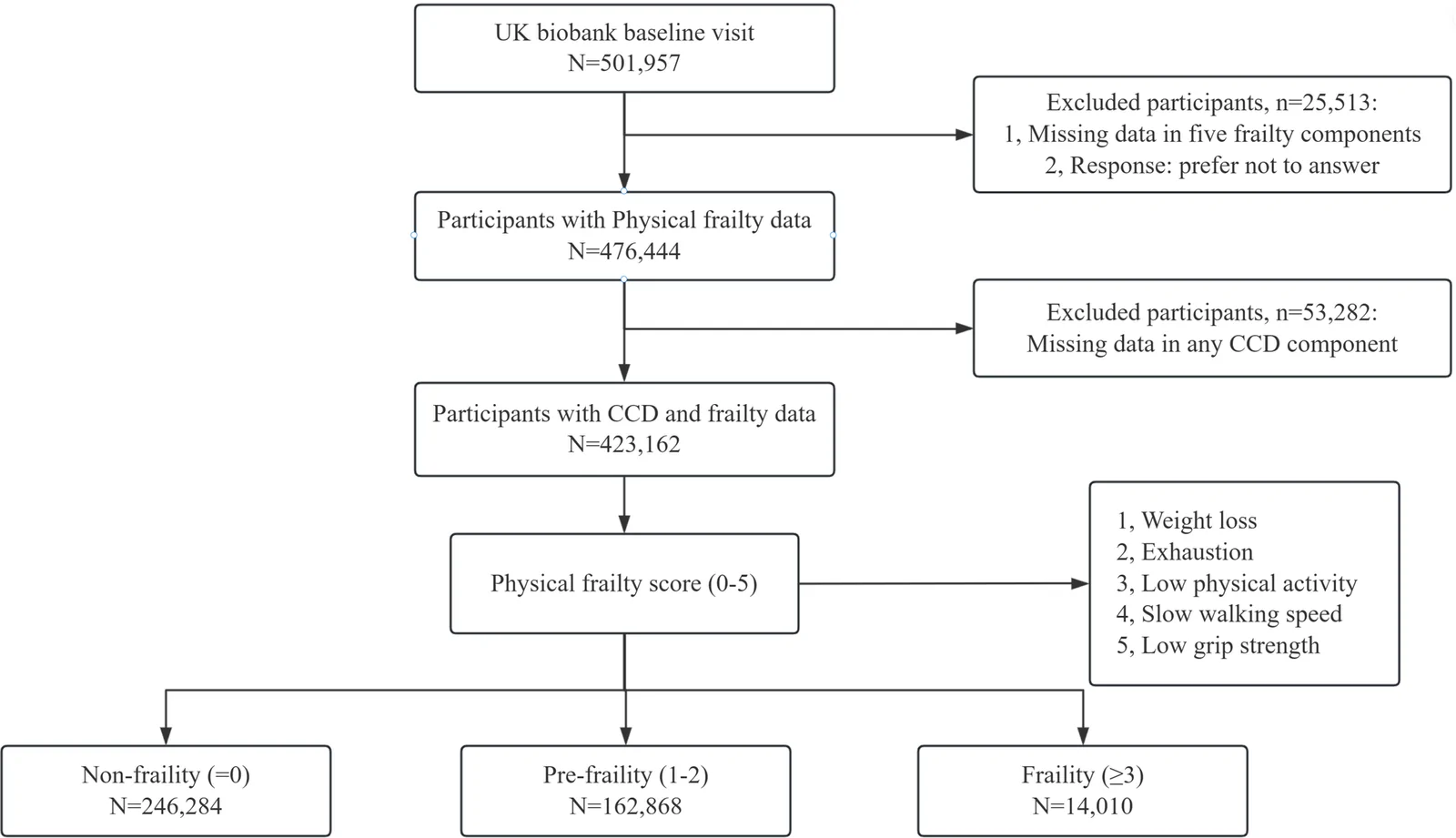
Study flow and distribution of the physical frailty status in the UK Biobank analytic cohort.

### Assessment of physical frailty

Physical frailty was assessed at baseline using a modified Fried phenotype adapted to UK Biobank data (10, 11). Five components were considered: weight loss, exhaustion, low physical activity, slow walking pace, and low grip strength. Each component was coded as present (1 point) or absent (0 points), yielding a total score ranging from 0 to 5. Participants were classified as non-frail (score 0), prefrail (scores 1–2), or frail (score ≥3). Because the UK Biobank does not reproduce all measurements from the original Fried phenotype, the exposure was described as a modified Fried phenotype.

### Outcome ascertainment

The primary outcome was incident CCD. The prespecified secondary outcomes were severe conduction block and left bundle branch or fascicular block. CCD was defined to include atrioventricular block, bundle branch block, fascicular block, non-specific intraventricular block, and other conduction disorders recorded in linked health records. Secondary outcomes included a severe conduction disease composite and left bundle branch or fascicular block recorded during the follow-up. The severe conduction outcome composite was defined as the first occurrence of a third-degree atrioventricular block [International Classification of Diseases, 10th Revision (ICD-10): I44.2] or a pacemaker-related procedure recorded during the follow-up. Pacemaker-related procedures were identified using OPCS-4 codes K60.1–K60.9 and K61.1–K61.9 (Supplementary Table 9), consistent with the outcome framework used in the previous UK Biobank study of Life's Essential 8 and incident cardiac conduction disorders. These code series include pacemaker implantation, lead resiting, system renewal and removal, and other specified or unspecified pacemaker procedures. Because pacemaker procedures may be undertaken for indications other than advanced atrioventricular or intraventricular block, we additionally performed a sensitivity analysis restricted to third-degree atrioventricular block, excluding the pacemaker-related procedure component (4).

Diagnostic outcomes were identified using ICD-10 codes for atrioventricular block (I44.0–I44.3), bundle branch block and fascicular block (I44.4–I45.4), and other conduction disorders (I45.5, I45.8, and I45.9). Outcome and censoring information was obtained from linked UK Biobank hospital inpatient diagnostic records, procedure records, and death registry records available in the data extraction performed on 4 September 2025, which served as the analytical cutoff for the primary analysis. Participants were followed from the date of baseline assessment until the first occurrence of the outcome of interest, death, loss to follow-up, or the analytical cutoff, whichever occurred first.

### Covariates

Covariates were selected based on the clinical relevance and previous epidemiological studies. Demographic variables included age, sex, and race. Socioeconomic variables included education level and the Townsend deprivation index (quartile 4 is the most deprived). Lifestyle variables included smoking status and alcohol consumption. Clinical covariates included hypertension, diabetes, dyslipidemia, body mass index (BMI), and estimated glomerular filtration rate (eGFR). Age was categorized as <65 or ≥65 years for subgroup analyses. Race was classified as White and other race. BMI was categorized as <25, 25–30, or >30 kg/m^2^, and eGFR was categorized as <60 or ≥60 mL/min/1.73 m^2^.

### Statistical analysis

All analyses were conducted using R version 4.4.2. Baseline characteristics were summarized according to the physical frailty status. Continuous variables were reported as mean (SD), and categorical variables were expressed as number (percentage). Group differences were assessed using analysis of variance for continuous variables and the chi-squared test for categorical variables.

Kaplan–Meier methods were used to estimate event-free survival according to the frailty category, and curves were compared using the log-rank test. Cox proportional hazards regression was used to estimate hazard ratios (HRs) and 95% confidence intervals (CIs), with the non-frail group as the reference. The crude model included no covariates. Model 1 was adjusted for age, sex, and race. Model 2 was additionally adjusted for education level, Townsend deprivation index, smoking status, alcohol consumption, hypertension, diabetes, dyslipidemia, BMI, and eGFR. Tests for trend were performed by modeling frailty categories as an ordinal variable. The frailty score was additionally analyzed as an ordered exposure to estimate the HR per one-point increment. The proportional hazards assumption was evaluated using scaled Schoenfeld residuals. Variable-specific and global tests were examined for each fully adjusted Cox model together with graphical inspection of the scaled Schoenfeld residuals over the follow-up time. No evidence of violation of the proportional hazards assumption was observed.

Missing covariate data were handled via complete-case analysis. Participants with missing values for any covariate required in a given model were excluded from that model, and no statistical imputation was performed. Consequently, the analytic sample size varied across models. For overall CCD, the crude model, model 1, and model 2 included 423,160 participants with 18,067 events, 421,811 participants with 17,998 events, and 390,949 participants with 16,576 events, respectively. The corresponding numbers were 423,160 participants with 10,652 events, 421,811 participants with 10,609 events, and 390,949 participants with 9,816 events for severe conduction block and were 423,160 participants with 5,710 events, 421,811 participants with 5,690 events, and 390,949 participants with 5,244 events for left bundle branch or fascicular block. Missingness for individual variables and the model-specific numbers of participants and events are reported in Supplementary Tables 1, 2, respectively.

Dose–response associations between the physical frailty score and conduction outcomes were examined using restricted cubic spline functions incorporated into multivariable Cox proportional hazards models. Because the physical frailty score had six discrete values ranging from 0 to 5, four knots were placed at scores 1–4 using the data-dependent knot-selection procedure implemented in the rms/Hmisc packages. Scores 1 and 4 served as the boundary knots, and scores 2 and 3 served as the internal knots. A frailty score of 0 was used as the reference. Separate models were fitted for overall CCD, the severe conduction outcome composite, and left bundle branch or fascicular block. The models were adjusted for age, sex, race, education level, Townsend deprivation index, smoking status, alcohol consumption, hypertension, diabetes, dyslipidemia, BMI, and eGFR. The overall association was evaluated using a Wald test of all spline terms, whereas non-linearity was assessed by jointly testing the non-linear spline terms against the null hypothesis that their coefficients were equal to zero. Adjusted HRs and 95% CIs were plotted across the observed range of the frailty score.

Subgroup analyses were conducted according to age, sex, race, education level, Townsend deprivation index, smoking status, alcohol consumption, hypertension, diabetes, dyslipidemia, BMI, and eGFR. Potential effect modification was assessed by including a multiplicative interaction term between frailty status and each subgroup variable. These analyses were exploratory, and no adjustment was made for multiple comparisons. Accordingly, all *P*-values for interaction were nominal, and the subgroup findings were interpreted as hypothesis-generating rather than as confirmatory.

Sensitivity analyses were performed by excluding outcome events occurring within the first 2 years of follow-up to reduce the potential influence of reverse causation. An additional analysis excluded participants who died during the follow-up to examine the robustness of the associations among survivors. The definitions and results of these sensitivity analyses are reported in Supplementary Tables 4, 5. All statistical tests were two-sided, and *P* < 0.05 was considered statistically significant. Because the completeness of records beyond the recommended censoring dates may vary across data sources and geographic regions, we repeated the Cox regression analyses with 30 September 2021 as a uniform administrative censoring date. The exposure definitions, covariate adjustment, and complete-case approach were otherwise identical to those used in the primary analysis.

## Results

### Baseline characteristics

The analysis included 423,162 participants, of whom 246,284 (58.2%) were non-frail, 162,868 (38.5%) were prefrail, and 14,010 (3.3%) were frail (Figure 1). Overall, 337,448 participants (79.7%) were less than 65 years of age, 231,845 (54.8%) were women, and 400,847 (95.0% of participants with available race data) were White (Table 1).

**Table 1:** Baseline characteristics according to the physical frailty status.

Characteristics		Participants, *N*				
			Physical frailty score			
		Total	Non-frailty (=0)	Prefrailty (1–2)	Frailty (≥3)	*P*-value
Participants [*N* (%)]		423,162 (100.0)	246,284 (100.0)	162,868 (100.0)	14,010 (100.0)	
Age (years)	<65	337,448 (79.7)	198,042 (80.4)	128,626 (79.0)	10,780 (76.9)	<0.001
	≥65	85,714 (20.3)	48,242 (19.6)	34,242 (21.0)	3,230 (23.1)	
Sex [*N* (%)]	Female	231,845 (54.8)	133,073 (54.0)	91,410 (56.1)	7,362 (52.5)	<0.001
	Male	191,317 (45.2)	113,211 (46.0)	71,458 (43.9)	6,648 (47.5)	
Race [*N* (%)]	White	400,847 (95.0)	237,672 (96.8)	150,887 (93.0)	12,288 (88.2)	<0.001
	Other race	20,966 (5.0)	7,936 (3.2)	11,389 (7.0)	1,641 (11.8)	
Education level [*N* (%)]	College or university degree	133,080 (31.8)	86,201 (35.3)	44,705 (27.9)	2,174 (15.9)	<0.001
	A levels/AS levels or equivalent	46,439 (11.1)	28,505 (11.7)	16,843 (10.5)	1,091 (8.0)	
	O levels/General Certificate of Secondary Education (GCSEs) or equivalent	90,515 (21.6)	53,327 (21.8)	34,691 (21.7)	2,497 (18.2)	
	Other education level	148,082 (35.4)	76,442 (31.3)	63,716 (39.8)	7,924 (57.9)	
Townsend deprivation index	Quartile 1	106,058 (25.1)	69,011 (28.0)	35,362 (21.7)	1,685 (12.0)	<0.001
	Quartile 2	106,233 (25.1)	66,074 (26.9)	37,989 (23.4)	2,170 (15.5)	
	Quartile 3	105,875 (25.1)	61,258 (24.9)	41,382 (25.4)	3,235 (23.1)	
	Quartile 4	104,484 (24.7)	49,692 (20.2)	47,894 (29.5)	6,898 (49.3)	
Smoking status [*N* (%)]	Never	226,804 (53.8)	136,951 (55.8)	83,920 (51.8)	5,933 (42.7)	<0.001
	Former	149,730 (35.5)	87,115 (35.5)	57,782 (35.6)	4,833 (34.8)	
	Current	45,157 (10.7)	21,570 (8.8)	20,452 (12.6)	3,135 (22.6)	
Drinking status [*N* (%)]	Never	18,255 (4.3)	7,818 (3.2)	8,962 (5.5)	1,475 (10.6)	<0.001
	Former	15,620 (3.7)	6,261 (2.5)	7,701 (4.7)	1,658 (11.9)	
	Current	388,882 (92.0)	232,106 (94.3)	145,957 (89.8)	10,819 (77.5)	
Hypertension [*N* (%)]	Yes	234,129 (55.3)	130,921 (53.2)	93,645 (57.5)	9,563 (68.3)	<0.001
	No	189,033 (44.7)	115,363 (46.8)	69,223 (42.5)	4,447 (31.7)	
Diabetes[ *N* (%)]	Yes	31,144 (7.4)	11,715 (4.8)	16,273 (10.0)	3,156 (22.5)	<0.001
	No	392,018 (92.6)	234,569 (95.2)	146,595 (90.0)	10,854 (77.5)	
Dyslipidemia[ *N* (%)]	Yes	244,780 (57.8)	137,971 (56.0)	97,009 (59.6)	9,800 (70.0)	<0.001
	No	178,382 (42.2)	108,313 (44.0)	65,859 (40.4)	4,210 (30.0)	
BMI (kg/m^2^)	<25	136,385 (32.3)	89,402 (36.3)	44,056 (27.1)	2,927 (21.1)	<0.001
	25–30	179,999 (42.6)	106,796 (43.4)	68,308 (42.1)	4,895 (35.4)	
	>30	106,125 (25.1)	50,086 (20.3)	50,020 (30.8)	6,019 (43.5)	
eGFR (mL/min/1.73 m^2^)	<60	6,514 (1.6)	2,847 (1.2)	3,041 (2.0)	626 (4.8)	
	≥60	391,952 (98.4)	230,271 (98.8)	149,369 (98.0)	12,312 (95.2)	
Systolic blood pressure (mmHg)		138.02 ± 18.66	138.37 ± 18.66	137.56 ± 18.63	137.17 ± 19.00	<0.001
Diastolic blood pressure (mmHg)		82.25 ± 10.13	82.41 ± 10.05	82.07 ± 10.21	81.71 ± 10.65	<0.001
Blood glucose (mmol/L)		5.13 ± 1.26	5.06 ± 1.02	5.21 ± 1.45	5.60 ± 2.20	<0.001
HbA1c (%)		5.46 ± 0.62	5.41 ± 0.51	5.52 ± 0.71	5.80 ± 1.04	<0.001
HDL-C (mmol/L)		1.45 ± 0.38	1.48 ± 0.38	1.41 ± 0.37	1.30 ± 0.37	<0.001
Triglycerides (mmol/L)		1.76 ± 1.03	1.71 ± 1.00	1.80 ± 1.06	2.02 ± 1.20	<0.001
Total cholesterol (mmol/L)		5.69 ± 1.15	5.77 ± 1.12	5.59 ± 1.17	5.32 ± 1.27	<0.001
LDL-C (mmol/L)		3.55 ± 0.87	3.60 ± 0.85	3.50 ± 0.89	3.31 ± 0.95	<0.001

Baseline cardiometabolic and behavioral characteristics differed across frailty categories (Table 1). Compared with non-frail participants, frail participants were more likely to be current smokers (22.6% vs. 8.8%) and to have hypertension (68.3% vs. 53.2%), diabetes (22.5% vs. 4.8%), BMI >30 kg/m^2^ (43.5% vs. 20.3%), and eGFR <60 mL/min/1.73 m^2^ (4.8% vs. 1.2%). The prevalence of these characteristics was generally intermediate in the prefrail group.

During the follow-up, 18,067 participants developed overall CCD, 10,652 experienced the severe conduction outcome composite, and 5,710 developed a left bundle branch or fascicular block. Crude event proportions increased across frailty categories for all three outcomes (Table 2).

**Table 2:** Incident cardiac conduction outcomes and outcome-specific follow-up according to the baseline physical frailty status. Follow-up durations and event counts correspond to the primary analysis using linked records available through the analytical cutoff date of 4 September 2025. The analytical cutoff represents the date of data extraction and should be distinguished from UK Biobank–recommended source- and region-specific censoring dates. Results with 30 September 2021 as a uniform administrative censoring date are presented in Supplementary Table 6.

Outcome or follow-up measure	Overall (*N* = 423,162)	Nonfrail (*n* = 246,284)	Prefrail (*n* = 162,868)	Frail (*n* = 14,010)	*P*-value
Overall cardiac conduction disorder					
Incident events [*n* (%)]	18,067 (4.3)	8,874 (3.6)	7,991 (4.9)	1,202 (8.6)	<0.001
Follow-up time (months)	196.15 ± 22.80	196.97 ± 21.00	195.33 ± 24.21	191.37 ± 33.13	<0.001
Severe conduction outcome composite					
Incident events [*n* (%)]	10,652 (2.5)	5,550 (2.3)	4,509 (2.8)	593 (4.2)	<0.001
Follow-up time (months)	197.61 ± 19.36	198.07 ± 18.23	197.11 ± 20.31	195.46 ± 25.84	<0.001
Left bundle branch or fascicular block					
Incident events [*n* (%)]	5,710 (1.3)	2,731 (1.1)	2,593 (1.6)	386 (2.8)	<0.001
Follow-up time (months)	198.79 ± 15.49	199.17 ± 14.48	198.35 ± 16.33	197.23 ± 21.16	<0.001

### Physical frailty and CCD during the follow-up

During the follow-up, 18,067 participants had a recorded CCD event. Events occurred in 8,874 of 246,284 participants who were non-frail (3.6%), 7,991 of 162,868 participants who were prefrail (4.9%), and 1,202 of 14,010 participants who were frail (8.6%). Kaplan–Meier curves showed lower event-free survival with increasing frailty category (log-rank *P* < 0.0001; Figure 2A).

**Figure 2:**
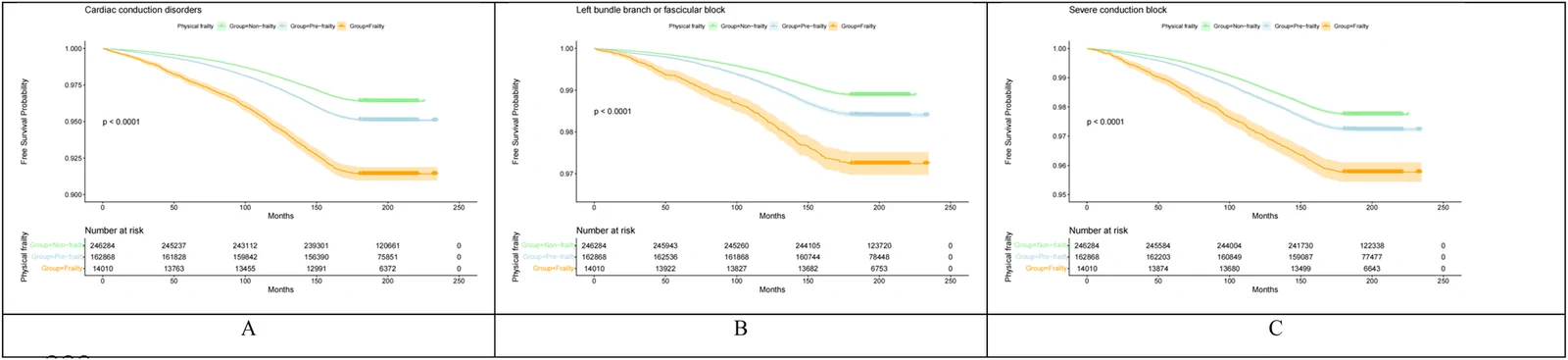
Kaplan–Meier curves for cardiac conduction disorder outcomes according to the physical frailty status. **(A)** Cardiac conduction disorders. **(B)** Left bundle branch or fascicular block. **(C)** Severe conduction block.

Relative to non-frailty, the crude HRs for CCD were 1.37 (95% CI, 1.33–1.41) for prefrailty and 2.46 (95% CI, 2.32–2.61) for frailty. After adjustment for demographic, socioeconomic, behavioral, and clinical covariates, the corresponding HRs were 1.23 (95% CI, 1.19–1.27) and 1.71 (95% CI, 1.60–1.83), respectively (*P* for trend <0.01; Table 3). Each one-point increase in frailty score was associated with an adjusted HR of 1.19 (95% CI, 1.17–1.21). Restricted cubic spline analysis showed an overall association between frailty score and CCD (*P* < 0.001) without strong evidence of departure from linearity (*P* for non-linearity=0.061; Figure 3A).

**Table 3:** Associations of physical frailty with cardiac conduction disorder outcomes.

	HR (95% CI)					
	Per 1-point increase in frailty score	*P*-value	Physical frailty score			
			Non-frailty (=0)	Prefrailty (1–2)	Frailty (≥3)	*P* trend
Cardiac conduction disorders						
CCD/total	18,067/423,160^a^		8,874/246,284	7,991/162,868	1,202/14,010	
Crude	1.31 (1.30–1.34)	<0.01	1 (ref.)	1.37 (1.33–1.41)	2.46 (2.32–2.61)	<0.01
Model 1	1.31 (1.29–1.33)	<0.01	1 (ref.)	1.38 (1.34–1.42)	2.37 (2.23–2.52)	<0.01
Model 2	1.19 (1.17–1.21)	<0.01	1 (ref.)	1.23 (1.19–1.27)	1.71 (1.60–1.83)	<0.01
Severe conduction block						
SCB/total	10,652/423,160^a^		5,550/246,284	4,509/162,868	593/14,010	
Crude	1.21 (1.19–1.24)	<0.01	1 (ref.)	1.23 (1.19–1.28)	1.90 (1.75–2.07)	<0.01
Model 1	1.20 (1.18–1.23)	<0.01	1 (ref.)	1.24 (1.19–1.29)	1.82 (1.67–1.99)	<0.01
Model 2	1.12 (1.10–1.15)	<0.01	1 (ref.)	1.13 (1.09–1.18)	1.44 (1.32–1.59)	<0.01
Left bundle branch or fascicular block						
LBB/total	5,710/423,160^a^		2,731/246,284	2,593/162,868	386/14,010	
Crude	1.34 (1.30–1.37)	<0.01	1 (ref.)	1.44 (1.36–1.52)	2.51 (2.26–2.79)	<0.01
Model 1	1.33 (1.29–1.36)	<0.01	1 (ref.)	1.44 (1.36–1.52)	2.42 (2.18–2.70)	<0.01
Model 2	1.18 (1.14–1.21)	<0.01	1 (ref.)	1.24 (1.17–1.32)	1.61 (1.43–1.81)	<0.01

Crude: No adjustments were made.

Model 1: Adjusted for age, sex, and race.

Model 2: Adjustment variables included: age, sex, race, education level, Townsend deprivation index, smoking status, drinking status, hypertension, diabetes, dyslipidemia, BMI, and eGFR.

The *P* for trend was calculated by treating the different frailty groups as a continuous variable in the models.

a Two participants with missing outcome-specific follow-up time were excluded from the crude models. Model-specific numbers of participants and events in the Statistical Analysis section, Table 3, and Supplementary Table 2.

**Figure 3:**
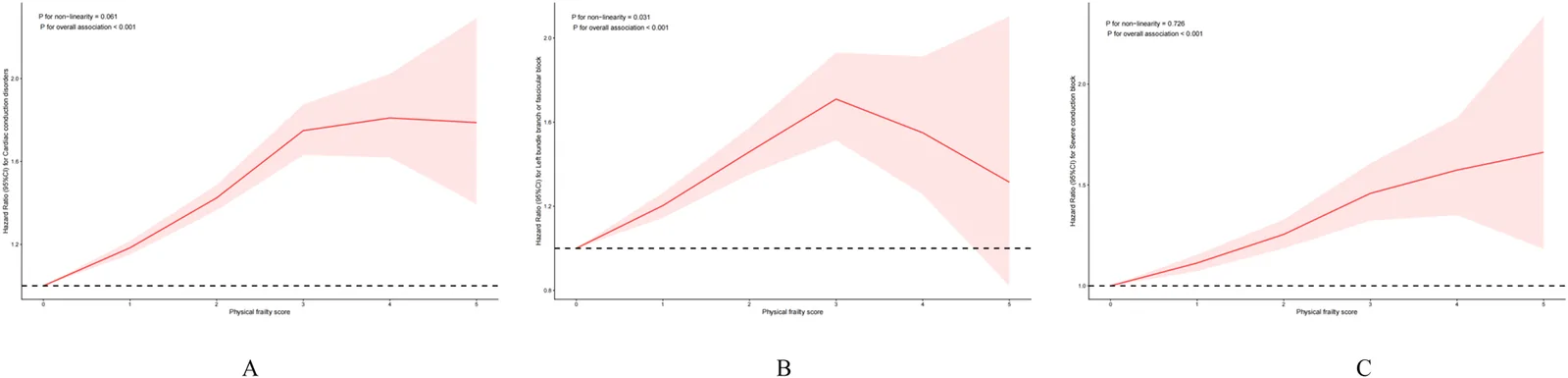
Dose-response associations between the physical frailty score and cardiac conduction disorder outcomes. **(A)** Cardiac conduction disorders. **(B)** Left bundle branch or fascicular block. **(C)** Severe conduction block.

### Secondary conduction outcomes

Left bundle branch or fascicular block occurred in 5,710 participants (Table 2), and event-free survival decreased across increasing frailty categories (log-rank *P* < 0.0001; Figure 2B). Compared with participants who were non-frail, the fully adjusted HR was 1.24 (95% CI, 1.17–1.32) for participants who were prefrail and was 1.61 (95% CI, 1.43–1.81) for participants who were frail (*P* for trend <0.01; Table 3). The adjusted HR per one-point increase in frailty score was 1.18 (95% CI, 1.14–1.21). Restricted cubic spline analysis provided modest evidence of non-linearity (*P* for non-linearity=0.031; Figure 3B), although estimates at the highest frailty scores were imprecise.

The severe conduction outcome composite occurred in 10,652 participants (Table 2), with lower event-free survival across increasing frailty categories (log-rank *P* < 0.0001; Figure 2C). The fully adjusted HR was 1.13 (95% CI, 1.09–1.18) for participants who were prefrail and was 1.44 (95% CI, 1.32–1.59) for participants who were frail (*P* for trend <0.01; Table 3). Each one-point increase in frailty score was associated with an adjusted HR of 1.12 (95% CI, 1.10–1.15). Restricted cubic spline analysis showed an overall association (*P* < 0.001) without evidence of departure from linearity (*P* for non-linearity=0.726; Figure 3C).

### Subgroup analyses

For CCD, adjusted associations with frailty were present across age, sex, and race strata (Table 4). Nominal evidence of interaction by age was observed (*P* for interaction <0.01): the adjusted HR for frailty vs. non-frailty was 1.86 (95% CI, 1.70–2.02) among participants who were less than 65 years of age and was 1.49 (95% CI, 1.34–1.66) among those who were ≥65 years of age. The nominal interaction *P*-values for sex and race were 0.92 and 0.50, respectively.

**Table 4:** Subgroup analyses of the associations between physical frailty and cardiac conduction disorder outcomes: (A) cardiac conduction disorders.

Subgroups		HR 95% CI					
		Per 1-point increase in frailty score	*P*-value	Physical frailty score			
				Non-frailty (=0)	Prefrailty (1–2)	Frailty (≥3)	*P* interaction
Age, years							<0.01
	<65	1.21 (1.18–1.24)	<0.01	1 (ref.)	1.25 (1.19–1.30)	1.86 (1.70–2.02)	
	≥65	1.15 (1.12–1.18)	<0.01	1 (ref.)	1.19 (1.13–1.25)	1.49 (1.34–1.66)	
Sex [*N* (%)]							0.92
	Female	1.19 (1.16–1.23)	<0.01	1 (ref.)	1.25 (1.18–1.32)	1.70 (1.51–1.91)	
	Male	1.18 (1.16–1.21)	<0.01	1 (ref.)	1.21 (1.17–1.26)	1.73 (1.59–1.88)	
Race [*N* (%)]							0.5
	White	1.19 (1.17–1.21)	<0.01	1 (ref.)	1.22 (1.18–1.26)	1.70 (1.58–1.82)	
	Other Race	1.20 (1.11–1.29)	<0.01	1 (ref.)	1.33 (1.11–1.60)	1.97 (1.52–2.56)	
Education level [*N* (%)]							0.98
	College or University degree	1.18 (1.13–1.23)	<0.01	1 (ref.)	1.16 (1.09–1.24)	1.79 (1.51–2.13)	
	A levels/AS levels or equivalent	1.17 (1.10–1.24)	<0.01	1 (ref.)	1.24 (1.12–1.38)	1.72 (1.33–2.22)	
	O levels/GCSEs or equivalent	1.18 (1.13–1.23)	<0.01	1 (ref.)	1.21 (1.12–1.29)	1.65 (1.40–1.95)	
	other education level	1.19 (1.16–1.22)	<0.01	1 (ref.)	1.26 (1.20–1.33)	1.72 (1.58–1.88)	
Townsend deprivation index							0.88
	Quartile 1	1.19 (1.15–1.24)	<0.01	1 (ref.)	1.25 (1.17–1.33)	1.53 (1.26–1.86)	
	Quartile 2	1.20 (1.16–1.25)	<0.01	1 (ref.)	1.23 (1.16–1.31)	1.70 (1.44–2.00)	
	Quartile 3	1.18 (1.14–1.22)	<0.01	1 (ref.)	1.18 (1.11–1.26)	1.77 (1.55–2.03)	
	Quartile 4	1.18 (1.14–1.21)	<0.01	1 (ref.)	1.24 (1.17–1.33)	1.74 (1.57–1.93)	
Smoking status [*N* (%)]							<0.01
	Never	1.22 (1.18–1.25)	<0.01	1 (ref.)	1.27 (1.21–1.33)	1.82 (1.63–2.03)	
	Former	1.17 (1.14–1.20)	<0.01	1 (ref.)	1.18 (1.13–1.24)	1.64 (1.48–1.82)	
	Current	1.16 (1.11–1.22)	<0.01	1 (ref.)	1.23 (1.12–1.36)	1.72 (1.46–2.02)	
Drinking status [*N* (%)]							0.12
	Never	1.17 (1.09–1.26)	<0.01	1 (ref.)	1.29 (1.10–1.52)	1.63 (1.27–2.10)	
	Former	1.17 (1.10–1.24)	<0.01	1 (ref.)	1.20 (1.03–1.40)	1.71 (1.39–2.11)	
	Current	1.19 (1.17–1.21)	<0.01	1 (ref.)	1.22 (1.18–1.26)	1.71 (1.59–1.85)	
Hypertension [*N* (%)]							<0.01
	Yes	1.19 (1.17–1.22)	<0.01	1 (ref.)	1.26 (1.21–1.31)	1.73 (1.60–1.86)	
	No	1.15 (1.11–1.20)	<0.01	1 (ref.)	1.13 (1.06–1.21)	1.70 (1.45–1.99)	
Diabetes [*N* (%)]							0.78
	Yes	1.19 (1.15–1.24)	<0.01	1 (ref.)	1.17 (1.07–1.28)	1.76 (1.56–2.00)	
	No	1.18 (1.16–1.21)	<0.01	1 (ref.)	1.23 (1.19–1.28)	1.66 (1.52–1.80)	
Dyslipidemia [*N* (%)]							0.04
	Yes	1.19 (1.16–1.21)	<0.01	1 (ref.)	1.24 (1.19–1.28)	1.67 (1.55–1.80)	
	No	1.19 (1.15–1.23)	<0.01	1 (ref.)	1.20 (1.13–1.27)	1.90 (1.64–2.20)	
BMI (kg/m^2^)							0.21
	<25	1.20 (1.15–1.25)	<0.01	1 (ref.)	1.23 (1.14–1.31)	1.90 (1.61–2.24)	
	25–30	1.18 (1.15–1.21)	<0.01	1 (ref.)	1.21 (1.15–1.27)	1.77 (1.58–1.98)	
	>30	1.18 (1.15–1.21)	<0.01	1 (ref.)	1.24 (1.17–1.31)	1.61 (1.46–1.78)	
eGFR (mL/min/1.73 m^2^)							0.31
	<60	1.23 (1.15–1.31)	<0.01	1 (ref.)	1.41 (1.18–1.67)	2.03 (1.59–2.58)	
	≥60	1.18 (1.16–1.20)	<0.01	1 (ref.)	1.22 (1.18–1.26)	1.70 (1.58–1.82)	

Across the additional exploratory strata, nominal interactions for CCD were observed by smoking status (*P* < 0.01), hypertension (*P* < 0.01), and dyslipidemia (*P* = 0.04; Table 4). For severe conduction block, nominal interactions were observed by age, sex, and smoking status (all *P* < 0.01; Supplementary Table 7). For left bundle branch or fascicular block, nominal interactions were observed by age (*P* = 0.02), smoking status (*P* < 0.01), drinking status (*P* = 0.02), hypertension (*P* = 0.04), and eGFR (*P* = 0.03; Supplementary Table 8). Estimates were less precise in smaller subgroups, particularly among participants of non-White race and those with eGFR <60 mL/min/1.73 m^2^.

### Sensitivity analyses

In the analysis restricted to third-degree atrioventricular block, the adjusted HR was 1.20 (95% CI, 1.09–1.32) for prefrailty and was 1.57 (95% CI, 1.28–1.92) for frailty (Supplementary Table 3). After exclusion of events occurring within the first 2 years of follow-up, the adjusted HRs for frailty vs. non-frailty were 1.68 (95% CI, 1.56–1.80) for overall CCD, 1.38 (95% CI, 1.25–1.52) for the severe conduction outcome composite, and 1.59 (95% CI, 1.41–1.80) for the left bundle branch or fascicular block (Supplementary Table 4). In the survivor-restricted analysis excluding participants who died during the follow-up, the corresponding HRs were 1.66 (95% CI, 1.51–1.81), 1.51 (95% CI, 1.34–1.70), and 1.52 (95% CI, 1.29–1.80), respectively (Supplementary Table 5). In the sensitivity analysis with 30 September 2021 as a uniform administrative censoring date, 14,691 overall CCD events, 9,019 severe conduction outcome events, and 4,685 left bundle branch or fascicular block events were recorded. The corresponding mean outcome-specific follow-up durations were 150.14, 150.80, and 151.43 months, respectively. In the fully adjusted complete-case models, the HRs comparing frailty with non-frailty were 1.82 (95% CI, 1.69–1.96) for overall CCD, 1.50 (95% CI, 1.36–1.66) for the severe conduction outcome composite, and 1.69 (95% CI, 1.49–1.92) for the left bundle branch or fascicular block. These estimates were consistent in direction and overall interpretation with those from the primary analysis (Supplementary Table 6).

## Discussion

In this large prospective cohort of UK Biobank participants, physical frailty was associated with a graded increase in CCD events recorded during the follow-up. The association was present in the prefrailty stage, became stronger among participants who were frail, and extended to the severe conduction disease composite and left bundle branch or fascicular block. These findings suggest that physical frailty may identify a subgroup with higher conduction disease risk before overt functional decline is fully established.

This study extends recent prevention-oriented work on conduction disease (2–5). Prior studies have linked CCD with cardiovascular health metrics, lifestyle factors, inflammation, and biological ageing markers (3–7). Our findings shift the focus of this literature from individual risk factors toward an integrated functional phenotype. This distinction is clinically relevant because frailty can be assessed using simple physical and questionnaire-based measures (10, 11), and recent UK Biobank evidence suggests that frailty may add risk information across cardiovascular–kidney–metabolic states (12). However, its incremental predictive value for conduction outcomes still requires formal evaluation.

The recent UK Biobank study by Zhang et al. (16) examined the relationship between physical frailty and incident arrhythmias, focusing primarily on atrial fibrillation and considering conduction system diseases as one of several secondary arrhythmia categories. The prevalence of prefrailty and frailty in the study was 37.6% and 3.5%, respectively, which closely matches the corresponding proportions of 38.5% and 3.3% in our cohort. They identified 12,901 incident conduction system disease events and reported an adjusted HR of 1.20 for prefrailty and that of 1.56 for frailty, with an HR of 1.15 per additional frailty component. Our corresponding estimates for overall CCD were 1.23, 1.71, and 1.19, respectively. Thus, the two studies provide closely aligned evidence for a graded association between physical frailty and conduction disease. The somewhat larger estimates in our study may reflect differences in outcome definitions, analytic eligibility criteria, covariate adjustment, and follow-up rather than a fundamentally different association. Our study extends the earlier observation by focusing specifically on conduction disease, including 18,067 CCD events, and by separately evaluating the left bundle branch or fascicular block and a severe conduction outcome composite. The latter findings were supported by an analysis restricted to third-degree atrioventricular block, in which the adjusted HRs were 1.20 for prefrailty and 1.57 for frailty, indicating that the association with the severe composite was not explained solely by pacemaker procedures.

The primary findings were supported by several sensitivity analyses. After exclusion of events occurring during the first 2 years of follow-up, the adjusted HRs for frailty vs. non-frailty were 1.68 for overall CCD, 1.38 for the severe conduction outcome composite, and 1.59 for the left bundle branch or fascicular block, compared with 1.71, 1.44, and 1.61, respectively, in the primary analyses. Associations also persisted in analyses excluding participants who died during the follow-up. The sensitivity analysis applying a uniform administrative censoring date of 30 September 2021 yielded directionally consistent estimates, supporting the robustness of the main findings to differences in the periods of data availability.

Strengths of this study include its large prospective cohort; substantial number of incident conduction events; assessment of clinically distinct conduction outcomes; and consistent results across categorical, per-point, and sensitivity analyses. The direct comparison with the earlier UK Biobank arrhythmia study also shows that the association between frailty and conduction disease is reproducible under related but non-identical outcome and model specifications. Therefore, the contribution of this study is a more focused characterization and stratification of this association rather than the first identification of a relationship between frailty and conduction disease.

The association observed at the prefrailty stage requires cautious interpretation. It does not demonstrate that reversing prefrailty would prevent conduction disease. Rather, frailty may reflect cumulative functional and cardiometabolic vulnerability not completely represented by individual baseline covariates. Nevertheless, residual confounding and shared antecedent disease remain possible, and the present study did not evaluate biological mediation.

Exploratory subgroup analyses showed generally similar directions of association across participant characteristics. Nominal evidence of interaction by age suggested a larger relative association in participants who were less than 65 years of age than in older participants. One possible interpretation is that frailty conveys more relative risk information when the background incidence of age-related conduction disease is lower (18, 19). However, the subgroup analyses involved multiple comparisons, were not adjusted for multiplicity, and included relatively small numbers in some categories. Therefore, all interaction *P*-values were nominal, and these findings should be considered as hypothesis-generating rather than as evidence of established effect modification.

Nonetheless, this study has several limitations that require consideration. First, the observational design precludes causal inference, and residual or unmeasured confounding cannot be excluded. Second, frailty and covariates were measured at baseline only. Changes in frailty, cardiovascular risk factors, incident comorbidities, medications, and treatment practices during the prolonged follow-up were not captured. In addition, medical management and diagnostic practices have changed during the follow-up period, potentially affecting outcome detection and the interpretation of baseline exposures in relation to long-term events. Third, outcomes were identified mainly from linked hospital diagnosis and procedure records. Subclinical, asymptomatic, or exclusively outpatient conduction abnormalities may have been missed, and differences in healthcare contact could have influenced case ascertainment. Fourth, pacemaker implantation is not specific to atrioventricular or intraventricular conduction block and may be performed for other indications, including sinus node dysfunction. Accordingly, the severe conduction outcome composite may include some events not directly attributable to the severe conduction block. The analysis restricted to third-degree atrioventricular block produced consistent results, but it does not eliminate possible outcome misclassification in the primary composite. Fifth, excluding events occurring in the first 2 years reduced concern about reverse causation but could not exclude subclinical conduction disease present for longer periods before diagnosis. Sixth, the analysis excluding deceased participants was not equivalent to a Fine–Gray competing-risk analysis and may have introduced survivor selection bias, and therefore, competing mortality remains a limitation. Seventh, missing covariate data were handled using complete-case analysis. Although the fully adjusted models retained approximately 92.4% of the baseline cohort, participants with complete data may have differed systematically from those excluded, and selection bias related to missingness cannot be ruled out. Eighth, the modified Fried phenotype included self-reported components and was assessed only once, introducing potential exposure misclassification. Ninth, UK Biobank participants are healthier and less socioeconomically and ethnically diverse than the general population. The small number of participants in some non-White and clinically impaired subgroups limited precision and may restrict generalizability. Finally, the primary analysis used linked records available at the time of data extraction, including records beyond the recommended source- and region-specific censoring dates of the UK Biobank. Because the completeness of these later records may vary across data sources and geographic regions, some events may have been incompletely ascertained. Nevertheless, the associations remained directionally consistent in the sensitivity analysis with 30 September 2021 as a uniform administrative censoring date.

In conclusion, physical frailty was associated with CCD recorded during the follow-up and with clinically relevant conduction disease subtypes in the UK Biobank. The results replicate the broader association between frailty and conduction system disease reported previously while providing more focused estimates for overall CCD, the left bundle branch or fascicular block, and severe conduction outcomes. Physical frailty may serve as a pragmatic marker of conduction disease vulnerability, but its incremental predictive value, causal relevance, and potential role in prevention require external validation, longitudinal assessment, and analyses that more fully account for competing mortality and changes in health status over time.

## Code availability statement

The analysis code is available from the corresponding author upon reasonable request, subject to institutional and UK Biobank data-use requirements.

## Data Availability

The data supporting the findings of this study are available from the UK Biobank under application no. 866163. Access is subject to approval by the UK Biobank and completion of a data access agreement. Researchers may apply for access through the UK Biobank Access Management System at https://www.ukbiobank.ac.uk/use-our-data/apply-for-access/.
