# Supplementary material for: Association of physical frailty with cardiac conduction disorder events in the UK Biobank
Source: Front Cardiovasc Med. 2026 Oct 19;13:1967667. doi: 10.3389/fcvm.2026.1967667 (PMC13724336; doi:10.3389/fcvm.2026.1967667)
Supplement: Supplementary file 1 [file Table7.docx]

**Supplementary Materials**

*Association of Physical Frailty With Cardiac Conduction Disorder Events in the UK Biobank*

| Subgroups |  | HR 95% CI | | | | | |
| --- | --- | --- | --- | --- | --- | --- | --- |
|  |  | Per 1-point increase in frailty score | P value | Physical frailty score | | |  |
|  |  |  |  | Non-frailty (=0) | Pre-frailty (1-2) | Frailty (>=3) | P interaction |
| Age, years |  |  |  |  |  |  | < 0.01 |
|  | <65 | 1.15 (1.12-1.19) | < 0.01 | 1(ref.) | 1.16 (1.10-1.23) | 1.54 (1.37-1.73) |  |
|  | >=65 | 1.07 (1.03-1.11) | < 0.01 | 1(ref.) | 1.09 (1.02-1.16) | 1.28 (1.10-1.49) |  |
| Sex, N(%) |  |  |  |  |  |  | < 0.01 |
|  | Female | 1.17 (1.12-1.21) | < 0.01 | 1(ref.) | 1.18 (1.10-1.27) | 1.61 (1.37-1.90) |  |
|  | Male | 1.11 (1.08-1.14) | < 0.01 | 1(ref.) | 1.11 (1.06-1.17) | 1.38 (1.23-1.55) |  |
| Race, N(%) |  |  |  |  |  |  | 0.69 |
|  | White | 1.12 (1.10-1.15) | < 0.01 | 1(ref.) | 1.13 (1.09-1.18) | 1.42 (1.29-1.57) |  |
|  | Other Race | 1.14 (1.04-1.26) | < 0.01 | 1(ref.) | 1.11 (0.89-1.39) | 1.65 (1.17-2.31) |  |
| Education level, N(%) |  |  |  |  |  |  | 0.3 |
|  | College or University degree | 1.12 (1.06-1.18) | < 0.01 | 1(ref.) | 1.10 (1.01-1.19) | 1.52 (1.21-1.93) |  |
|  | A levels/AS levels or equivalent | 1.07 (0.98-1.16) | 0.13 | 1(ref.) | 1.04 (0.90-1.20) | 1.46 (1.02-2.09) |  |
|  | O levels/GCSEs or equivalent | 1.16 (1.10-1.23) | < 0.01 | 1(ref.) | 1.14 (1.04-1.25) | 1.62 (1.30-2.01) |  |
|  | other education level | 1.12 (1.08-1.15) | < 0.01 | 1(ref.) | 1.17 (1.10-1.24) | 1.40 (1.23-1.58) |  |
| Townsend deprivation index |  |  |  |  |  |  | 0.98 |
|  | Quartile 1 | 1.16 (1.10-1.22) | < 0.01 | 1(ref.) | 1.20 (1.11-1.30) | 1.46 (1.14-1.88) |  |
|  | Quartile 2 | 1.11 (1.06-1.16) | < 0.01 | 1(ref.) | 1.10 (1.02-1.20) | 1.33 (1.06-1.68) |  |
|  | Quartile 3 | 1.13 (1.08-1.18) | < 0.01 | 1(ref.) | 1.12 (1.03-1.22) | 1.56 (1.29-1.88) |  |
|  | Quartile 4 | 1.11 (1.07-1.16) | < 0.01 | 1(ref.) | 1.11 (1.02-1.21) | 1.42 (1.22-1.64) |  |
| Smoking status, N(%) |  |  |  |  |  |  | < 0.01 |
|  | Never | 1.13 (1.09-1.17) | < 0.01 | 1(ref.) | 1.16 (1.09-1.23) | 1.43 (1.23-1.67) |  |
|  | Former | 1.15 (1.11-1.19) | < 0.01 | 1(ref.) | 1.13 (1.06-1.21) | 1.62 (1.42-1.85) |  |
|  | Current | 1.04 (0.98-1.11) | 0.22 | 1(ref.) | 1.04 (0.90-1.19) | 1.09 (0.84-1.40) |  |
| Drinking status, N(%) |  |  |  |  |  |  | 0.61 |
|  | Never | 1.12 (1.02-1.23) | 0.02 | 1(ref.) | 1.03 (0.83-1.28) | 1.49 (1.06-2.08) |  |
|  | Former | 1.09 (1.01-1.19) | 0.04 | 1(ref.) | 1.12 (0.91-1.39) | 1.33 (0.99-1.79) |  |
|  | Current | 1.12 (1.10-1.15) | < 0.01 | 1(ref.) | 1.14 (1.09-1.19) | 1.44 (1.30-1.60) |  |
| Hypertension, N(%) |  |  |  |  |  |  | 0.54 |
|  | Yes | 1.12 (1.09-1.15) | < 0.01 | 1(ref.) | 1.14 (1.09-1.20) | 1.42 (1.28-1.58) |  |
|  | No | 1.12 (1.07-1.18) | < 0.01 | 1(ref.) | 1.11 (1.02-1.21) | 1.56 (1.25-1.94) |  |
| Diabetes, N(%) |  |  |  |  |  |  | 0.68 |
|  | Yes | 1.12 (1.07-1.18) | < 0.01 | 1(ref.) | 1.14 (1.02-1.28) | 1.41 (1.19-1.69) |  |
|  | No | 1.12 (1.09-1.15) | < 0.01 | 1(ref.) | 1.13 (1.08-1.18) | 1.46 (1.30-1.63) |  |
| Dyslipidemia, N(%) |  |  |  |  |  |  | 0.1 |
|  | Yes | 1.12 (1.10-1.15) | < 0.01 | 1(ref.) | 1.13 (1.07-1.18) | 1.45 (1.31-1.61) |  |
|  | No | 1.12 (1.06-1.17) | < 0.01 | 1(ref.) | 1.15 (1.06-1.25) | 1.36 (1.08-1.71) |  |
| BMI, kg/m^2 |  |  |  |  |  |  | 0.34 |
|  | <25 | 1.13 (1.08-1.20) | < 0.01 | 1(ref.) | 1.15 (1.06-1.26) | 1.51 (1.19-1.91) |  |
|  | 25-30 | 1.13 (1.09-1.17) | < 0.01 | 1(ref.) | 1.14 (1.08-1.22) | 1.41 (1.20-1.66) |  |
|  | >30 | 1.11 (1.07-1.15) | < 0.01 | 1(ref.) | 1.10 (1.02-1.18) | 1.41 (1.23-1.61) |  |
| eGFR, mL/min/1.73m² |  |  |  |  |  |  | 0.62 |
|  | <60 | 1.13 (1.02-1.24) | 0.01 | 1(ref.) | 1.31 (1.05-1.65) | 1.42 (1.00-2.04) |  |
|  | >=60 | 1.12 (1.10-1.15) | < 0.01 | 1(ref.) | 1.13 (1.08-1.17) | 1.46 (1.33-1.61) |  |

Table S7. Subgroup analyses of the association between physical frailty and severe conduction block.
