# Supplementary material for: Association of physical frailty with cardiac conduction disorder events in the UK Biobank
Source: Front Cardiovasc Med. 2026 Oct 19;13:1967667. doi: 10.3389/fcvm.2026.1967667 (PMC13724336; doi:10.3389/fcvm.2026.1967667)
Supplement: Supplementary file 2 [file Table8.docx]

**Supplementary Materials**

*Association of Physical Frailty With Cardiac Conduction Disorder Events in the UK Biobank*

| Subgroups |  | HR 95% CI | | | | | |
| --- | --- | --- | --- | --- | --- | --- | --- |
|  |  | Per 1-point increase in frailty score | P value | Physical frailty score | | |  |
|  |  |  |  | Non-frailty (=0) | Pre-frailty (1-2) | Frailty (>=3) | P interaction |
| Age, years |  |  |  |  |  |  | 0.02 |
|  | <65 | 1.17 (1.13-1.22) | < 0.01 | 1(ref.) | 1.22 (1.13-1.32) | 1.61 (1.38-1.88) |  |
|  | >=65 | 1.18 (1.13-1.23) | < 0.01 | 1(ref.) | 1.26 (1.16-1.38) | 1.57 (1.31-1.89) |  |
| Sex, N(%) |  |  |  |  |  |  | 0.07 |
|  | Female | 1.15 (1.10-1.21) | < 0.01 | 1(ref.) | 1.23 (1.12-1.34) | 1.45 (1.19-1.77) |  |
|  | Male | 1.19 (1.15-1.24) | < 0.01 | 1(ref.) | 1.25 (1.16-1.35) | 1.69 (1.46-1.97) |  |
| Race, N(%) |  |  |  |  |  |  | 0.6 |
|  | White | 1.18 (1.14-1.21) | < 0.01 | 1(ref.) | 1.24 (1.17-1.31) | 1.59 (1.41-1.80) |  |
|  | Other Race | 1.21 (1.06-1.38) | < 0.01 | 1(ref.) | 1.35 (0.96-1.88) | 1.84 (1.15-2.95) |  |
| Education level, N(%) |  |  |  |  |  |  | 0.52 |
|  | College or University degree | 1.17 (1.09-1.26) | < 0.01 | 1(ref.) | 1.20 (1.07-1.35) | 1.48 (1.06-2.08) |  |
|  | A levels/AS levels or equivalent | 1.25 (1.13-1.39) | < 0.01 | 1(ref.) | 1.36 (1.13-1.64) | 1.97 (1.28-3.04) |  |
|  | O levels/GCSEs or equivalent | 1.12 (1.04-1.20) | < 0.01 | 1(ref.) | 1.04 (0.92-1.18) | 1.46 (1.10-1.93) |  |
|  | other education level | 1.19 (1.14-1.24) | < 0.01 | 1(ref.) | 1.34 (1.23-1.46) | 1.67 (1.43-1.95) |  |
| Townsend deprivation index |  |  |  |  |  |  | 0.25 |
|  | Quartile 1 | 1.20 (1.12-1.29) | < 0.01 | 1(ref.) | 1.28 (1.14-1.44) | 1.34 (0.93-1.93) |  |
|  | Quartile 2 | 1.22 (1.14-1.30) | < 0.01 | 1(ref.) | 1.23 (1.10-1.38) | 1.80 (1.37-2.37) |  |
|  | Quartile 3 | 1.17 (1.10-1.24) | < 0.01 | 1(ref.) | 1.17 (1.04-1.30) | 1.77 (1.41-2.21) |  |
|  | Quartile 4 | 1.15 (1.09-1.21) | < 0.01 | 1(ref.) | 1.29 (1.15-1.46) | 1.53 (1.27-1.85) |  |
| Smoking status, N(%) |  |  |  |  |  |  | < 0.01 |
|  | Never | 1.21 (1.15-1.27) | < 0.01 | 1(ref.) | 1.28 (1.18-1.40) | 1.70 (1.40-2.07) |  |
|  | Former | 1.16 (1.11-1.22) | < 0.01 | 1(ref.) | 1.17 (1.07-1.27) | 1.50 (1.25-1.80) |  |
|  | Current | 1.17 (1.08-1.26) | < 0.01 | 1(ref.) | 1.40 (1.18-1.67) | 1.79 (1.35-2.38) |  |
| Drinking status, N(%) |  |  |  |  |  |  | 0.02 |
|  | Never | 1.11 (0.99-1.24) | 0.08 | 1(ref.) | 1.42 (1.09-1.83) | 1.19 (0.75-1.87) |  |
|  | Former | 1.11 (1.00-1.23) | 0.05 | 1(ref.) | 0.97 (0.74-1.26) | 1.44 (1.01-2.04) |  |
|  | Current | 1.19 (1.15-1.23) | < 0.01 | 1(ref.) | 1.24 (1.17-1.32) | 1.66 (1.45-1.89) |  |
| Hypertension, N(%) |  |  |  |  |  |  | 0.04 |
|  | Yes | 1.19 (1.15-1.23) | < 0.01 | 1(ref.) | 1.30 (1.21-1.38) | 1.62 (1.42-1.85) |  |
|  | No | 1.12 (1.04-1.20) | < 0.01 | 1(ref.) | 1.07 (0.94-1.20) | 1.63 (1.22-2.17) |  |
| Diabetes, N(%) |  |  |  |  |  |  | 0.9 |
|  | Yes | 1.18 (1.11-1.25) | < 0.01 | 1(ref.) | 1.21 (1.04-1.41) | 1.59 (1.28-1.98) |  |
|  | No | 1.18 (1.14-1.22) | < 0.01 | 1(ref.) | 1.25 (1.17-1.33) | 1.58 (1.37-1.84) |  |
| Dyslipidemia, N(%) |  |  |  |  |  |  | 0.07 |
|  | Yes | 1.19 (1.15-1.23) | < 0.01 | 1(ref.) | 1.29 (1.21-1.38) | 1.59 (1.39-1.81) |  |
|  | No | 1.15 (1.08-1.23) | < 0.01 | 1(ref.) | 1.11 (0.99-1.24) | 1.76 (1.35-2.29) |  |
| BMI, kg/m^2 |  |  |  |  |  |  | 0.41 |
|  | <25 | 1.17 (1.09-1.26) | < 0.01 | 1(ref.) | 1.23 (1.08-1.39) | 1.71 (1.28-2.30) |  |
|  | 25-30 | 1.18 (1.12-1.24) | < 0.01 | 1(ref.) | 1.23 (1.13-1.34) | 1.69 (1.38-2.06) |  |
|  | >30 | 1.18 (1.12-1.23) | < 0.01 | 1(ref.) | 1.26 (1.14-1.40) | 1.52 (1.27-1.81) |  |
| eGFR, mL/min/1.73m² |  |  |  |  |  |  | 0.03 |
|  | <60 | 1.33 (1.19-1.47) | < 0.01 | 1(ref.) | 1.67 (1.24-2.26) | 2.59 (1.75-3.83) |  |
|  | >=60 | 1.17 (1.13-1.20) | < 0.01 | 1(ref.) | 1.23 (1.16-1.30) | 1.54 (1.35-1.75) |  |

Table S8. Subgroup analyses of the association between physical frailty and left bundle branch or fascicular block.
