# Supplementary material for: Association of physical frailty with cardiac conduction disorder events in the UK Biobank
Source: Front Cardiovasc Med. 2026 Oct 19;13:1967667. doi: 10.3389/fcvm.2026.1967667 (PMC13724336; doi:10.3389/fcvm.2026.1967667)
Supplement: Supplementary file 3 [file Table9.docx]

**Supplementary Materials**

*Association of Physical Frailty With Cardiac Conduction Disorder Events in the UK Biobank*

**Table S9. OPCS-4 procedure codes used to identify pacemaker-related procedures**

| OPCS-4 code | Procedure description |
| --- | --- |
| K60.1 | Implantation of intravenous cardiac pacemaker system, not elsewhere classified |
| K60.2 | Resiting of lead of intravenous cardiac pacemaker system |
| K60.3 | Renewal of intravenous cardiac pacemaker system |
| K60.4 | Removal of intravenous cardiac pacemaker system |
| K60.5 | Implantation of intravenous single-chamber cardiac pacemaker system |
| K60.6 | Implantation of intravenous dual-chamber cardiac pacemaker system |
| K60.7 | Implantation of intravenous biventricular cardiac pacemaker system |
| K60.8 | Other specified cardiac pacemaker system introduced through vein |
| K60.9 | Unspecified cardiac pacemaker system introduced through vein |
| K61.1 | Implantation of cardiac pacemaker system, not elsewhere classified |
| K61.2 | Resiting of lead of cardiac pacemaker system, not elsewhere classified |
| K61.3 | Renewal of cardiac pacemaker system, not elsewhere classified |
| K61.4 | Removal of cardiac pacemaker system, not elsewhere classified |
| K61.5 | Implantation of single-chamber cardiac pacemaker system |
| K61.6 | Implantation of dual-chamber cardiac pacemaker system |
| K61.7 | Implantation of biventricular cardiac pacemaker system |
| K61.8 | Other specified cardiac pacemaker system |
| K61.9 | Unspecified cardiac pacemaker system |

OPCS-4, Office of Population Censuses and Surveys Classification of Interventions and Procedures, Fourth Revision. In the primary analysis, the severe conduction outcome composite was defined as the first occurrence of third-degree atrioventricular block (ICD-10 I44.2) or a pacemaker-related procedure identified using the OPCS-4 K60.1–K60.9 or K61.1–K61.9 code series. Because these series include pacemaker implantation, lead resiting, system renewal, and removal, this component was interpreted as a record of clinically relevant pacemaker-related intervention rather than as an indication-specific measure of pacemaker implantation.
